# Analysis of pre-hospital consultations with Crowd Medical Services at Premier League Football Club Fulham (2004-2013): planning for the future

**DOI:** 10.1186/1757-7241-23-S2-A17

**Published:** 2015-09-11

**Authors:** Ruth H Bird, Tim Nuttall

**Affiliations:** 1 Barts and the London NHS Trust, London, UK; 2 Event Medicine Company, Aldershot, UK

## Background

Craven Cottage has seen plans approved for a new riverside stand, raising capacity to 30,000 seats.

## Aims and method

The aim of this study was to examine the usage of Crowd Medical Services to plan for increased capacity. We completed a retrospective data analysis (2004-2013) to assess whether the number or type of injuries requiring medical assistance has any correlation to crowd numbers.

## Results

Over 9 seasons, there were 830 presentations to medical services, 512 new injuries/trauma and 318 medical presentations. There was one fatal non-traumatic cardiac arrest and 19 assaults.

43 patients were transported to hospital (5.18%).

## Discussion

There were 4,157,597 total crowd attendances at Fulham FC matches, with a consultation rate per 10,000 gate admissions of 1.97. This is comparable to previously published premiership data [1].

Analysis of the location of accidental injuries showed no statistical differences. More assaults occurred in areas where fans mix, irrespective of capacity.

This study has shown no significant differences or trends in trauma, medical cases or consultations per 10,000. (Chi square 9x2 contingency tables).

There was no statistical difference between crowd numbers and either injuries sustained or in assaults. Fulham's plans to increase capacity should not affect match to match provision of medical cover though it will be accounted for in major incident planning. The rate of consultation per 10,000 attendances has remained around 2 per 10,000 and has not been influenced by the increased crowd capacity or increases in attendance after gaining promotion or entry to the European cup competition. Crowd disturbances resulting in assault have remained at a low level and assaults do not seem to represent a threat to non-violent fans.
